# No New Relevant Treatment Options for L-DOPA-Induced Dyskinesia from a Clinician’s Point of View

**DOI:** 10.3390/neurolint18030059

**Published:** 2026-03-20

**Authors:** Thomas Müller

**Affiliations:** Department of Neurology, St. Joseph Hospital Berlin-Weißensee, Gartenstr. 1, 13088 Berlin, Germany; th.mueller@alexianer.de or thomas.mueller@ruhr-uni-bochum.de; Tel.: +49-30-92790223; Fax: +49-30-92790703

**Keywords:** dyskinesia, Parkinson’s disease, levodopa, amantadine

## Abstract

Background: The term dyskinesia describes involuntary movements of the face, body and extremities. Frequently, they appear following and in relation with prior oral long-lasting and high-dose levodopa therapy in Parkinson’s disease patients. Onset of these motion sequences causes patient distress and caregiver embarrassment with declined quality of life. Continuity of nigrostriatal postsynaptic dopamine receptor stimulation delays occurrence of dyskinesia. A pulsatile pattern with temporary too high dopamine receptor excitation promotes manifestation of dyskinesia. Methods: This narrative review describes past pharmacologic approaches for therapy of dyskinesia, such as the principle of continuous dopamine receptor stimulation. Discussion and Conclusions: Novel concepts were tested. They influenced neurotransmission of serotonin and altered stimulation of dopamine receptor subtypes. The translation of successful experimental research outcomes into valuable clinical trial results with consecutive approval of drugs with a new mode of action under the indication “antidyskinetic” repeatedly failed. An exception is the open-channel blocker of the N-methyl-D-aspartate receptor and dopamine reuptake inhibitor amantadine with its moderate dyskinesia-reducing effects, particularly in its extended-release formulation. This antiviral compound also improves impaired motor behavior and reduces “OFF” intervals. Therefore, amantadine is currently experiencing a certain resurgence in regions where its extended-release formulations are marketed for therapy of levodopa-induced dyskinesia.

## 1. Introduction

Parkinson’s disease (PD) is the second most frequent chronic neurodegenerative disorder worldwide. Currently its incidence ranges from 8 to 18 per 100,000 individuals. A doubling is assumed by 2030 [1]. Onset and diagnosis of PD are rare below the age of 50. There is an exponential increase after age 60. PD in fact reflects an entity of various subtypes of this disease. They resemble each other and are heterogeneous [2,3]. Generally, motor and non-motor symptoms and their progress are different in each individual patient. PD is mainly diagnosed as consequence of an initially transient, later permanent onset of at least two of the so-called “cardinal” motor features, bradykinesia, rigidity and resting tremor. It is estimated that they appear after an approximate 50–60% dopamine depletion as a result of loss of nigrostriatal dopamine-synthesizing neurons. This chronic neurodegenerative process is responsible for the manifestation of impairment of motor behavior. Unspecific non-motor features, such as one- or both-sided pain syndromes with intermittent onset of muscle cramps, depression or apathy, are frequent, initial, unspecific signs of PD. Therefore, PD is often not recognized in the early so-called “prodromal stages”. Accordingly, clinical diagnosis often corresponds to a more advanced disease stage due to wrong interpretation and lack of awareness of early non-specific clinical symptoms [2,3].

## 2. Oral Levodopa Therapy Induces Motor Complications Sooner or Later

**Figure 1:**
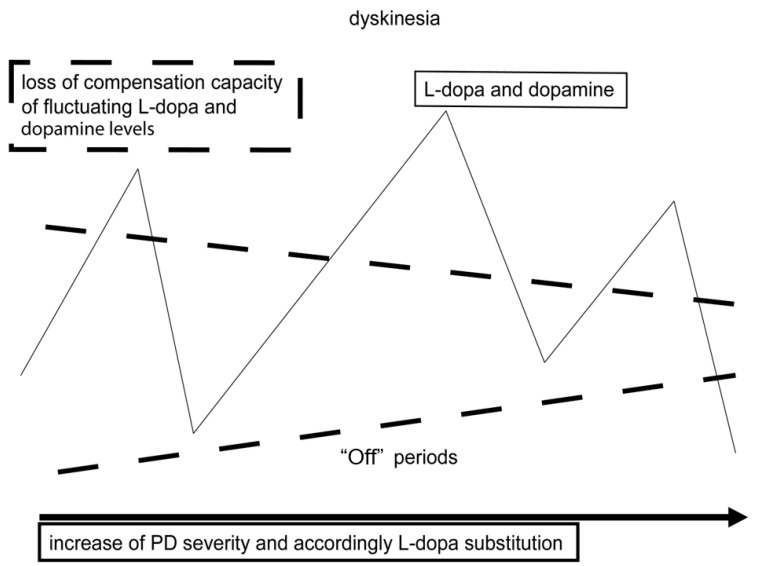
Hypothesis: compensation capacity of fluctuating L-dopa and dopamine is lost with advancement of Parkinson’s disease.

Oral levodopa (L-dopa) administration, in combination with an only peripherally acting dopa decarboxylase inhibitor (DDI), is efficacious. This combination has a relatively low side-effect profile in comparison with other dopamine-substituting agents, i.e., dopamine agonists. Therefore, its acceptance by patients is high for the clinical maintenance of PD patients. Following intake and duodenal absorption, L-dopa is transported through the intact blood–brain barrier in PD patients. In the brain, L-dopa is converted to dopamine by dopa decarboxylase mainly in presynaptic nigrostriatal and mesolimbic brain regions. Both are mainly involved in the onset and regulation of the severity of motor and non-motor features of PD [4]. Thus, the efficacy of oral L-dopa formulations also depends on peripheral gastrointestinal, mainly duodenal, absorption, the resulting plasma bioavailability and the subsequent transport to the brain. Amino acid transporter systems in the gastrointestinal tract and at the blood–brain barrier considerably influence the L-dopa effects. This scenario is additionally affected by the short plasma L-dopa half-life, which is prolonged to approximately 30–90 min by combination with a DDI. The variability of L-dopa plasma behavior is high and individually differs from patient to patient. Nutrition components, which contain or are metabolized to amino acids, i.e., from proteins, may further limit the transporter capacity [5,6]. These mechanisms additionally contribute to pulsatile plasma fluctuations of L-dopa. They are accordingly translated into a non-physiological, so-called “pulsatile” excitation of postsynaptic dopamine receptors following the conversion of L-dopa to dopamine in the brain by the enzyme dopa decarboxylase (Figure 1). It is believed that this stimulation pattern generates irregular postsynaptic gene and receptor function, which contributes to or even triggers onset of dyskinesia.

Both mechanisms may in turn alter downstream neuron activity with further neurotransmitter imbalances, i.e., of serotonin or glutamate [7,8,9,10,11,12]. There are individual differences in compensation capacity of these irregular stimulation patterns of postsynaptic dopamine receptors. The dopamine autoreceptor provides one mechanism to control presynaptic dopamine generation and release to the synaptic cleft to a certain extent. PD progress causes an increasing loss of presynaptic dopamine-synthesizing neurons. The compensatory capacity for compensation of central dopamine fluctuations is lost more and more (Figure 1). Presynaptic generation, storage and regulation of dopamine release for continuous, nigrostriatal postsynaptic dopamine receptor stimulation increasingly depends on further exogenously administered dopamine-substituting agents, such as inhibitors of monoamine oxidase-B (MAO-B), of catechol-O-methyltransferase (COMT) and of dopamine reuptake, like amantadine. At that moment, the honeymoon period of uncomplicated L-dopa therapy ends in a smoldering fashion [13,14,15]. Oral-L-dopa-associated motor complications, such as “wearing-off” phenomena, more intense “OFF” states or dyskinesia, slowly manifest. L-dopa itself is also increasingly metabolized to dopamine in serotonin-synthesizing (5-HT) neurons independent of dopamine-sensitive autoreceptor regulation [15]. This alternative conversion pathway promotes more pronounced fluctuations of dopamine levels in nigrostriatal and mesolimbic regions. At this stage PD patients experience changes in motor and non-motor behavior initially dependent and later independent of previous oral L-dopa intake as an important side effect of chronic oral L-dopa/DDI therapy [15,16,17,18,19].

### 2.1. The Benefit of L-Dopa on Motor Behavior Changes During PD Progression

Thus, L-dopa dosing and response reflects PD progress to a limited extent (for review: [20]). PD patients in early disease stages experience longer-lasting beneficial L-dopa effects. Mostly they only require an L-dopa intake three times daily. Then, with advance of PD, the duration of the clinical benefit, the so-called “motor response” derived from each oral L-dopa/DDI intake, becomes shorter. The decline of this therapeutic window provides shorter relief from impairment of motor behavior following oral application of L-dopa/DDI. At this stage, patients initially experience worsening of motor symptoms before the next oral L-dopa/DDI intake (Figure 1). This aforementioned, so-called “wearing-off” phenomenon is still predictable, because it is associated with the prior drug intake [20,21,22]. Further PD progress requires higher dosing of L-dopa. These motor complications predominantly resulting from oral L-dopa/DDI therapy become unpredictable and more intense [23,24]. The correlation with oral L-dopa/DDI administration increasingly becomes unpredictable.

### 2.2. Dyskinesia in PD Patients

Accordingly, PD progression with the demand for higher dosing of oral L-dopa/DDI increases the probability of an at least temporary too high stimulation of dopamine-sensitive structures in nigrostriatal and mesolimbic regions, particularly of postsynaptic receptors. Here a role of dopamine D_3_ receptors was discussed following experimental research in dyskinesia animal models of PD [14,25,26]. Particularly, too high dosing of dopamine-substituting compounds with short half-life, applied in so called “on-demand” therapies, may further promote temporary dyskinesia onset in clinical practice. However, to date, evidence is lacking from clinical investigations under randomized conditions or real-world analyses to support this hypothesis [27,28]. A further theory focuses on the onset of functional disturbances in the basal ganglia circuit, considering it responsible for the manifestation of motor complications. The theory postulates that loss of nigral dopamine-synthesizing neurons changes the interaction between the motor cortex and the striatum. It is believed that this procedure consequently contributes to onset of dyskinesia and “OFF” intervals. This model suggests deep brain stimulation (DBS) as a potent therapeutic approach. However, one has to consider that this surgical procedure may also contribute to a more continuous stimulation of postsynaptic dopaminergic neurons from the neurochemical point of view. An increase in endogenous dopamine synthesis and release was shown during DBS [29,30,31,32,33]. In detail, the upregulation of thalamocortical activity with consecutive suppression of the inhibitory output from the pallidum to the ventrolateral thalamus is still a hypothetical mode of action for alleviation of motor symptoms in PD only. In fact, the detailed mechanism of DBS of globus pallidum internum for the reduction in dyskinesia is not completely understood. Setting lesions in the medial globus pallidus also particularly improves dyskinesia [34]. Again, one assumes that DBS reduces the excitatory drive on thalamic and motor cortical nuclei [35,36,37]. However, DBS performance raises the risk for personality changes and cognitive disturbances even within six months [38]. Another older, still-hypothetical consideration suggests that loss of dopamine-producing neurons results in postsynaptic plastic changes with hypersensitivity of postsynaptic dopaminergic neurons [39,40,41]. Further existing hypotheses discuss the involvement of other neurotransmitter systems, such as glutamate, gamma-aminobutyric acid, serotonin, histamine, adenosine, and cannabinoids, in the development of L-dopa-induced dyskinesia (for review: [13,14,26]). These theories are further supported by experimental findings or are based on clinical observations [26]. They do not consider that sedative effects of drugs generally alleviate the severity of dyskinesia [42,43,44,45,46]. In summary, the pathogenesis of L-dopa-induced dyskinesia in PD patients is not fully understood in detail [26]. It is known that young, so-called “early-onset” PD patients with longer L-dopa lifetime exposure and/or higher oral L-dopa dosing obviously develop dyskinesia more frequently. This was observed in the 600 mg L-dopa arm of the “Levodopa and the Progression of Parkinson’s Disease” (L-DOPA) study and in the “Stalevo Reduction in Dyskinesia Evaluation in Parkinson’s Disease” (STRIDE-PD) trial but not in the “Levodopa in EArly Parkinson’s disease” (LEAP) study. However, the LEAP study exposed PD patients to lower L-dopa/DDI dosages only [47,48,49]. Individuals without PD, such as patients with restless leg syndrome, do not develop dyskinesia during long-term intake of L-dopa in contrast to PD patients. Probable reasons are an intact dopamine neurotransmission system and lower dosing of L-dopa, if applied.

### 2.3. Dyskinesia Phenomenology

Dependent on the dosing of dopamine substitution therapies with oral L-dopa/DDI formulations with and without inhibition of COMT and monoamine oxidase B (MAO-B), onset of motor (Figure 1) complications is mainly characterized by combined manifestation of dyskinesia and “OFF” states. Thus, they are related to the pharmacokinetic behavior of short-living dopamine-substituting compounds, such as L-dopa. “ON”-state dyskinesia occurs during an interval with maximum improvement from motor symptoms (“peak-dose” dyskinesia). “Diphasic” dyskinesia is also known. It occurs soon following L-dopa intake at the moment when the patient enters an “ON” interval or when the L-dopa response goes down and the “OFF” phase starts again. Accordingly, different kinds of dyskinesia exist in clinical practice. In contrast uniform forms are observed in animal models of dyskinesia only. Motion patterns are chorea, athetosis, dystonia, stereotypy, ballism or a combination of them in the clinical presentation of PD patients [50,51,52,53]. Accordingly, dyskinesia severity may be mild and “not troublesome” or even “troublesome” and thus completely disabling during both “ON” and “OFF” intervals [50,51,52,53]. Generally, dyskinesia manifestation may influence patients’ and caregivers’ quality of life. Patients themselves are frequently not aware of the presence of mild dyskinesia. Most PD patients prefer these phases with mild dyskinesia intervals in the “ON” state and dislike less dyskinesia within longer “OFF” time periods. Severe dyskinesia and dystonia-like muscle cramps resembling movement patterns may also cause considerable disability particularly in advanced PD patients [54]. At this disease stage, they may cause pain syndromes and sometimes even severe speech and swallowing problems. Accordingly, they may contribute to weight loss, which in turn contributes to higher L-dopa bioavailability as a result of an inverse relationship between body weight and L-dopa plasma levels [55,56,57]. Dyskinesia may even become exhausting or life-threatening accompanied by breathing problems, i.e., in the rare case of diaphragmatic dyskinesia onset [52,58]. Dyskinesia onset and appearance are also closely associated with exposure to stress or emotions. Generally, dyskinesia severity increases during interaction with nearly all kinds of stressors, i.e., emotional distress.

### 2.4. Current Therapy Possibilities for Dyskinesia in PD Patients

#### 2.4.1. Delay of Dyskinesia Onset

There is convincing evidence that continuous nigrostriatal postsynaptic dopamine receptor stimulation (CDS) ameliorates motor complications. Accordingly, dopamine agonists with their long half-life, or as an alternative, an intense, longer-lasting direct stimulation of postsynaptic dopamine uptake sites, may delay dyskinesia manifestation [59]. These pharmacological modes of action also reduce the need for oral L-dopa/DDI dosing. In the long term they decline the clinical consequences of pulsatile L-dopa brain delivery with subsequent fluctuating postsynaptic dopamine receptor stimulation (Figure 1).

#### 2.4.2. Device-Supported Approaches of Drug Administration

Subcutaneous infusions of apomorphine or foslevdopa, or intestinal infusion of L-dopa/carbidopa gel (LCIG) or plus entacapone (LECIGON^®^), may also reduce both “OFF” periods and dyskinesia by a pump device [60,61,62,63,64,65,66,67]. It enables a fine-tuning of drug transport to the brain in a more continuous manner even with intraventricular application [66,68,69]. All these infusion scenarios are cost-intensive and demand considerable support by caregivers due to the employed pump device. They may only be employed in suitable and thus selected PD patients. Therefore, availability of oral administration of an antidyskinetic compound would still be the optimal easy-to-perform treatment option for PD patients.

#### 2.4.3. Antidyskinetic Drugs

Glutamate (NMDA) antagonism is considered as one therapeutic drug treatment option against dyskinesia even with antagonists of metabotropic glutamate receptor type 5 [70,71,72,73,74]. It is based on the basal ganglia circuit model following experimental and neurosurgical findings and clinical study results, i.e., with amantadine and its NMDA-antagonistic properties (IC50: 38.9 µM in 5 µM NMDA) [75,76]. Accordingly, amantadine, particularly in its sustained-release formulations, was demonstrated as an efficacious drug in clinical trial outcomes. These compounds are approved with the label of “antidyskinetic” drug. Similar to DBS with its positive impact on dopamine synthesis and release, amantadine also influences neurotransmission of dopamine [77]. This old antiviral drug also has anticholinergic properties. Moreover, it also inhibits dopamine reuptake to presynaptic dopamine-generating neurons. Particularly, this mechanism contributes to a more intense and continuous dopamine neurotransmission at the synaptic cleft pharmacologically. One may conclude that this mechanism is the decisive factor responsible for the observed antidyskinetic effect in clinical studies with PD patients suffering from motor complications. Therefore, as to be expected, a reduction of “OFF” phases was also observed in these trials (for review: [78,79]).

### 2.5. Pharmacology of Novel Antidyskinetic Drugs

The unmet need still exists to introduce further therapeutic strategies for improvement of oral L-dopa/DDI-associated onset of dyskinesia in PD patients [54]. One compound is buspirone. It is a partial agonist at serotonin (5-HT) type 1A (5-HT1A) uptake sites. This compound also antagonizes dopamine D_2_ receptors [80,81]. Resembling compounds are DSP-9632P, which is administered via the skin as a patch, and befiradol [82,83,84]. They also act as 5-HT1A receptor agonists. JM-010 combines buspirone and zolmitriptan, and both together stimulate 5-HT type 1B and 5-HT type 1D receptors [80]. The 5-HT1B/1D agonist SKF-99101-H lowered efficacy of L-dopa in a PD primate model with 1-methyl-4-phenyl-1,2,3,6-tetrahydropyridine (MPTP)-caused lesions [85,86,87]. Ketamine mainly acts as a preferential N-methyl-D-aspartate (NMDA) antagonist. However, it also shows affinity for cholinergic receptors, 5-HT type 2A (5-HT2A) and dopamine D_2_ receptors [88,89,90,91]. Similar to amantadine, its primary mode of action is far from clear in detail. AV-101 (L-4-chlorokynurenine) antagonizes at the glycine-binding site of NMDA receptors [92,93,94]. The precise modes of action of dyskinesia-improving Tianqi Pingchan granules are also not known in detail. They influence G protein-coupled receptor kinase 6 expression (GRK6) [95,96]. Mesdopetam also showed some positive effects on dyskinesia. It acts as a dopamine D_3_/D_2_ antagonist, but also has agonist-like binding properties at dopamine receptors [80,97,98]. CPL500036 selectively inhibits phosphodiesterase 10A (PDE10A), lenrispodun constrains phosphodiesterase 1B (PDE1B), and both were also described as antidyskinetic after application in animal models [80,88] (Table 1). Lipoic acid application also showed antidyskinetic effects in experimental research. Further development with clinical studies was not initiated [99]. The various studies on all these compounds share multiple similarities. Mainly experimental researchers provided a large body of promising study results. The investigations were performed in PD-like uniform dyskinesia models [14]. Convincing clinical outcomes are often still lacking in phase III trials.

### 2.6. Efficacy in Clinical Studies

Buspirone finally showed negative outcomes also in the combination with zolmitriptan [80,81]. Tandospirone improved dyskinesia in some PD patients, but limited beneficial efficacy of L-dopa in a pilot clinical trial. A small phase IIa study with the 5-HT1a agonist NLX-112 reduced L-dopa-induced dyskinesia [82,83,84,100]. Ketamine improved severity of dyskinesia in a case series. Mesdopetam also showed some signals for an amelioration of L-dopa-induced dyskinesia in PD patients [97].

### 2.7. Conclusions

Many experimental and some clinical investigations are under way for the label to act as an antidyskinetic agent as a new indication. Positive clinical results will support authorities’ approval as a convincing drug therapy for PD patients on oral L-dopa/DDI therapy. To date, a breakthrough has not yet been achieved. Promising positive signals were found in some phase II studies with compounds. They mainly impact regulation of dopamine substitution and in particular 5-HT 1a receptor stimulation. Convincing phase III outcomes for their approval as an antidyskinetic compound are currently not available. Only extended-release amantadine formulations showed some antidyskinetic effects. They are not marketed worldwide, i.e., in Europe.

## 3. Discussion

Considerable efforts have been undertaken to develop a drug with the label of an “antidyskinetic” efficacy. Experimental researchers repeatedly reported initial promising outcomes. These investigations in animal PD models with L-dopa-induced dyskinesia were positive probably due to a standardized and therefore more uniform generation of dyskinesia. Then, translation into the clinic with positive phase III study results frequently failed. Probable reasons are the clinical heterogeneous presentation of L-dopa-induced dyskinesia and of the PD entity itself [64]. One further drawback was that the performed phase III studies did not standardize the current available therapeutic modes of dopamine substitution. Patients on oral L-dopa/DDI therapy alone or in combination with and without inhibitors of MAO-B and COMT and/or dopamine agonists were included. The performed study analyses did not stratify along the various pharmacological modes of action of dopamine substitution. Additionally, co-medication with sedative compounds, like certain antidepressants, neuroleptics, etc., may also influence the clinical presentation, the variability and the vulnerability for onset of oral L-dopa/DDI-induced dyskinesia in PD patients. A further still-underestimated factor even in clinical trials may also be the compliance of PD patients. They often have the tendency to change drug dosing, the intake regimen, or both. The current availability of existing “on-demand” therapies may hypothetically aggravate this situation. Too high dosing with intake of, i.e., soluble L-dopa/benserazide or sublingual apomorphine may further promote an at least temporary onset of dyskinesia in the real world of clinical practice and even in clinical trials.

It is well accepted that delay of dyskinesia may be achieved with CDS [64,65]. It is mainly enabled by dopamine agonists with their mostly longer half-life or by continuous brain delivery of dopamine-substituting compounds with short half-life, i.e., apomorphine or L-dopa. Accordingly, the available pump devices allow continuous brain delivery of the applied drug with additional fine-tuning of drug titration with adaptation to the patient’s needs. Compliance issues are reduced by this approach. Currently, subcutaneous infusion systems of apomorphine or foslevdopa/carbidopa and duodenal infusion of an L-dopa/DDI gel with and without the COMT inhibitor entacapone are available.

Generally, therapy for dyskinesia is complex and may be time consuming. Moreover, there is the considerable, well-known influence of high expectations implemented with a new therapy based on the reward model via the mesolimbic system. It resembles to the mechanisms of placebo application to a certain extent. Emotions and various stressors influence manifestation and severity of dyskinesia. This was particularly the case in the case of sarizotan with its 5-HT1a properties This compound was positive in phase II performed in selected highly specialized centers. Then, negative outcomes appeared in phase III with more widespread use worldwide [102,103,104]. Generally, treatment of L-dopa-induced dyskinesia also demands an individualized titration and co-medication with dopamine-substituting compounds in clinical practice. Therefore, standardized study conditions with enforced stable dosing of dopamine substitution, i.e., during the whole study interval, are questionable. Moreover, a novel antidyskinetic agent should also be easy to apply. In this regard it is noteworthy that the already approved “antidyskinetic” amantadine extended-release application is far more simple and cheaper than therapeutic alternatives like deep brain stimulation, pallidotomy or drug application with a pump device system. Moreover, the efficacy of generic available amantadine on dyskinesia is also well described. These results support amantadine use again. Amantadine was also discussed as a neuroprotective compound in the past. A lack of randomized studies was noted in terms of the antidyskinetic effects of amantadine immediate release (IR) [105]. These study results performed with amantadine IR did not receive interest and support from the pharmaceutical industry, the study world or the authorities with their demand for cost-intensive randomized clinical trials. Accordingly, a return on investment is unlikely without any approval as an innovative drug in terms of amantadine use. This was the motivation to develop amantadine as an extended-release formulation [75,76,106,107]. Thus, for approval and the label “antidyskinetic”, the trials were done with this old, well-known PD drug in a new pharmacokinetic environment. As aforementioned, amantadine also acts a dopamine reuptake inhibitor from the synaptic cleft. Therefore, amantadine also only supports the concept of continuous dopaminergic stimulation similar to dopamine agonists, infusion of dopamine-substituting compounds, DBS, or therapeutic metabolism slowing of L-dopa and dopamine with enzyme inhibitors [77,79,108].

From this point of view, the ideal pharmacological co-medication for a trial that investigates a new pharmacological principle for treatment of L-dopa-induced dyskinesia would be a more standardized PD drug regimen [88]. This concomitant dopamine substitution should combine central inhibition of MAO-B, peripheral and even central COMT inhibition and blocking of dopamine reuptake by amantadine. All these modes of action stabilize neurotransmission of dopamine. A more constant oral L-dopa brain delivery, enabled with IPX 203, would support this concept [109,110].

Future randomized clinical trials on antidyskinetic effects should include these considerations in their designs. Moreover, one should not underestimate heterogeneous profiles of enzymatic L-dopa degradation activity or adaptive changes in peripheral and central employed dopamine-substituting drug metabolism with varying capacity due to enzyme induction by, i.e., chronic L-dopa/DDI intake. Variability of gastrointestinal absorption and blood–brain barrier amino acid transporter function, particularly for L-dopa, may also interfere with a putative beneficial antidyskinetic drug effect over time in a randomized clinical trial.

Therefore, it would make sense to simplify and standardize at least oral L-dopa/DDI treatment in future clinical trials that aim to demonstrate an antidyskinetic effect in a randomized clinical trial with comparison against placebo. Influencing variables particularly on L-dopa metabolism should be minimized and standardized. To ease recruitment, the at least temporary worsening of the motor behavior of the study participants should be accepted by ethics committees. These aforementioned suggestions may hypothetically elevate the likelihood of positive study outcomes similar to uniform standardization procedures in experimental dyskinesia models in PD animal models. Similar procedures in terms of inclusion criteria and design approaches were already performed in the past in patients with wearing-off phenomena. Here a prior optimization of the dopamine substitution drug regimen was performed. This was a benefit for the patient and was therefore ethically approved. In terms of a clinical investigation of an antidyskinetic effect within a randomized clinical trial, the situation is different. The intention is to improve long-term side effects of oral L-dopa/DDI substitution. A precondition should be at least a certain intensity and daily duration of dyskinesia with a somehow uniform presentation of involuntary movement sequences. A future positive study outcome would be a therapeutic breakthrough for PD patients; one should only ask for one positive trial result for approval by authorities. Performance of the trial in a few experienced PD treatment centers should be enough with a limited number of study participants. They should be motivated to accept an at least temporary worsening of dyskinesia presentation by, i.e., elevation of L-dopa/DDI dosing during a distinct shorter study than usual nowadays. Nevertheless, the real world of clinical practice will decide whether it will employ such an antidyskinetic drug or not, following market authorization.

## 4. Conclusions

Heterogeneity exists in terms of dyskinesia onset, expression and course, similar to PD itself. The currently increasing complexity of clinical drug research with bureaucratic restrictions limits drug development in general in combination with drug-approving authorities’ aggravated general fear of side effects by patients. These factors contribute to the fact that, to date, no new, more generally valid treatment approach is likely to appear on the horizon for the treatment of dyskinesia in PD patients for use in clinical practice.

## Figures and Tables

**Table 1:** Examples of antidyskinetic compounds in clinical trials.

Compound	Main Target Mechanism	Current Clinical Trial Phase	Main Finding
Befiradol	Serotonin type 1A agonist	II	UDysRS total score decreased by 6.3 points compared with placebo (−2.4). UPDRS III scores decreased by 3.7 points, placebo (+0.1) [100]
Buspirone	Serotonin 1A agonist	III	Buspirone failed to improve significantly LID in PD patients UDysRS −5.5 [−19, +4] vs. −8.0 [−12, −3]
DSP-9632P	Serotonin 1A agonist	I	Safe, transdermal application
Ketamine	Hypothesis: Ketamine disrupts pathological interaction between motor cortex neurons [101]	Suspended	Case series
Mesdopetam	D2/D3 antagonist	IIb	Failed on the primary endpoint “good on time” (daily time spent without troublesome dyskinesia), severity of dyskinesia improved according to UDysRS [97]

Abbreviations: D2/D3, dopamine receptor 2/receptor 3; UDysRS, Unified Dyskinesia Rating Scale; UPDRS III, Unified Parkinson’s Disease Rating Scale part motor examination.

## Data Availability

No new data were created or analyzed in this study.

## Abbreviations

The following abbreviations are used in this manuscript:

5-HT	Serotonin
CDS	Continuous dopaminergic stimulation
COMT	Catechol-O-methyltransferase
DBS	Deep brain stimulation
DDI	Dopa decarboxylase inhibitor
IR	Immediate release
LCIG	Levodopa carbidopa intestinal gel
L-dopa	Levodopa
MAO-B	Monoaminooxidase B
NMDA	Glutamate, N-methyl-D-Aspartate
PD	Parkinson’s disease
PDE	Phosphodiesterase
